# Altered Fecal Microbiota Correlates with Liver Biochemistry in Nonobese Patients with Non-alcoholic Fatty Liver Disease

**DOI:** 10.1038/srep32002

**Published:** 2016-08-23

**Authors:** Baohong Wang, Xiangyang Jiang, Min Cao, Jianping Ge, Qiongling Bao, Lingling Tang, Yu Chen, Lanjuan Li

**Affiliations:** 1 National Collaborative Innovation Center for Diagnosis and Treatment of Infectious Diseases, State Key Laboratory for Diagnosis and Treatment of Infectious Diseases, the First Affiliated Hospital, School of Medicine, Zhejiang University, Hangzhou, 310003, China

## Abstract

Increasing evidence suggests a role of intestinal dysbiosis in obesity and non-alcoholic fatty liver disease (NAFLD). But it remains unknown in nonobese NAFLD. This prospective, cross-sectional study sought to characterize differences in fecal microbiota between nonobese adult individuals with and without NAFLD and their potential association with metabolic markers of disease progression. A total of 126 nonobese subjects were enrolled: 43 NAFLD and 83 healthy controls (HC). The microbial community was profiled by denaturing gradient gel electrophoresis and examined by 454 pyrosequencing of the 16S ribosomal RNA V3 region. Lower diversity and a phylum-level change in the fecal microbiome were found in NAFLD. Compared with HC, patients had 20% more phylum *Bacteroidetes* (*p* = 0.005) and 24% less *Firmicutes* (*p* = 0.002). Within *Firmicutes*, four families and their 8 genera, which were short-chain fatty acids-producing and 7α-dehydroxylating bacteria, were significantly decreased. Moreover, Gram-negative (G−) bacteria were prevalent in NAFLD (*p* = 0.008). Furthermore, a significant correlation with metabolic markers was revealed for disturbed microbiota in NAFLD. This novel study indicated that intestinal dysbiosis was associated with nonobese NAFLD and might increase the risk of NAFLD progression.

Non-alcoholic fatty liver disease (NAFLD) is the most common chronic liver condition in Western countries^1^ and some Asian countries^2^. NAFLD, ranging from simple non-alcoholic fatty liver to non-alcoholic steatohepatitis (NASH)^3^, is considered to be the hepatic manifestation of metabolic syndrome. Without an effective available treatment, the prognosis of NASH is poor because of the risk of progressive liver diseases such as cirrhosis and hepatocellular carcinoma. NAFLD is characterized by a broad spectrum of manifestations ranging from simple steatosis, NASH to liver cirrhosis and hepatocellular carcinoma^4^. Without an effective available treatment, the prognosis of NASH is poor. The human intestinal microbiota (IM) plays an important role in human health and diseases^5^,^6^,^7^ such as obesity^8^,^9^, diabetes mellitus^10^, liver cirrhosis^11^ and cancer^12^. Due to its close link with obesity, the pathogenesis of NAFLD has also been proposed to be the result of multiple ‘hits’, including the widely accepted contribution of the altered gut microbiota associated with obesity^13^,^14^.

Alterations of microbial communities, particularly a shift associated with obesity, correlates closely with the prevalence and progress of NAFLD^15^,^16^,^17^,^18^. For example, Zhu *et al*. has suggested a connection between gut-derived endogenous alcohol and NASH in the pathogenesis of obese children^18^. Raman *et al*. have revealed that a significant compositional shift in the IM (such as the decrease of some selected members of *Firmicutes*) is associated with NAFLD^17^. However, subsequent studies in adults have shown inconsistent and even contradictory results. For instance, Mouzaki M *et al*. have found a decrease in *Bacteroidetes* in obese patients with NASH compared with healthy controls (HCs) but no difference between patients with simple steatosis and HCs^16^. In these previous studies, all patients had a high body mass index (BMI) (>26), and the BMI and age of the NAFLD patients were significantly higher than those of HCs^16^,^17^. BMI could be a major confounder, because BMI has been demonstrated to be a major determinant of compositional changes to microbial communities^8^. To our knowledge, no studies have directly assessed the relationship between IM composition and nonobese adult patients with NAFLD.

Importantly, NAFLD is not rare in nonobese adults^19^,^20^,^21^,^22^,^23^. A recent study in Hong Kong showed that 20% of the nonobese population are NAFLD^23^. Another study in mainland China showed that the prevalence of NAFLD is 7.27% at baseline, and among these, 8.88% of nonobese subjects developed NAFLD during a 5-year follow-up^22^. In an adult population in Taiwan, the prevalence of NAFLD has been reported to be 11.5% in nonobese adults^19^. The study in Korea has reported a prevalence of NAFLD of 23.4% among 768 non-obese, nondiabetic individuals older than 30 years^21^. A study conducted in India has reported that 75% of the NAFLD patients studied had a BMI <25 Kg/m^2^, and 54% were neither overweight nor abdominal obesity^20^. Therefore, it is critically important to understand whether abnormal IM is associated with non-obese patients with NAFLD to better understand and prevent NAFLD.

Here, we used metagenomic approaches, including a bacterial community fingerprinting method of denaturing gradient gel electrophoresis (DGGE) and high-throughput 454 pyrosequencing targeting the 16S rRNA gene, to characterize the fecal microbial communities in nonobese adult individuals with NAFLD. Furthermore, to determine which of the thousands of microbial species are crucial for human health and to understand the potential molecular host-microbiome interactions, correlation analysis between the fecal microbiomic profiles and the clinical biochemical markers of NAFLD progression^22^ was also performed. Our findings provide a more comprehensive understanding of fecal microbiota in nonobese individuals with NAFLD and facilitate future preventive attempts to manipulate the commensal microbiota in nonobese patients with NAFLD.

## Results

### Clinical data of individuals in the nonobese cohort

Altogether, 126 nonobese subjects with (n = 43) or without (n = 83) NAFLD were enrolled in this prospective cross-sectional study. We performed anthropometric and biochemical phenotyping of the individuals (Table 1). Differences in clinical biochemical metabolic markers were compared between individuals with and without NAFLD. These markers are used to provide important information about liver function and to identify potential NAFLD patients. The liver synthetic function of all the individuals was in the normal range, as determined by albumin and International Normalized Ratio levels (data not shown). The age and gender distributions between the individuals with and without NAFLD were similar. All subjects consumed an omnivorous Chinese diet. Although all enrolled subjects were nonobese, the individuals with NAFLD had higher BMI, waist circumference, hip circumference and waist-to-hip ratio values (*p* = 0.004, 0.0001, 0.006, and 0001, respectively). In consistent with reports by Xu *et al*. of nonobese NAFLD^22^, the individuals with NAFLD were characterized by higher levels of alanine aminotransferase (ALT) (*p* < 0.0001), alkaline phosphatase (*p* = 0.002), total triglycerides (*p* < 0.0001), very low-density lipoprotein cholesterol (*p* < 0.0001), gamma-glutamyl transpeptidase (γ-GT) (*p* < 0.0001), and serum uric acid (*p* = 0.016). In addition, the patients had markedly lower levels of high-density lipoprotein cholesterol (*p* = 0.0003) compared with those of HCs. The increased levels of these markers suggest that individuals with NAFLD are featured by metabolic disturbances known to be associated with an increased risk of liver dysfunction and hepatocyte lipid accumulation and decreased β-oxidation energy production^24^.

### Differed pattern of fecal microbiota between individuals with and without NAFLD

**Figure 1:**
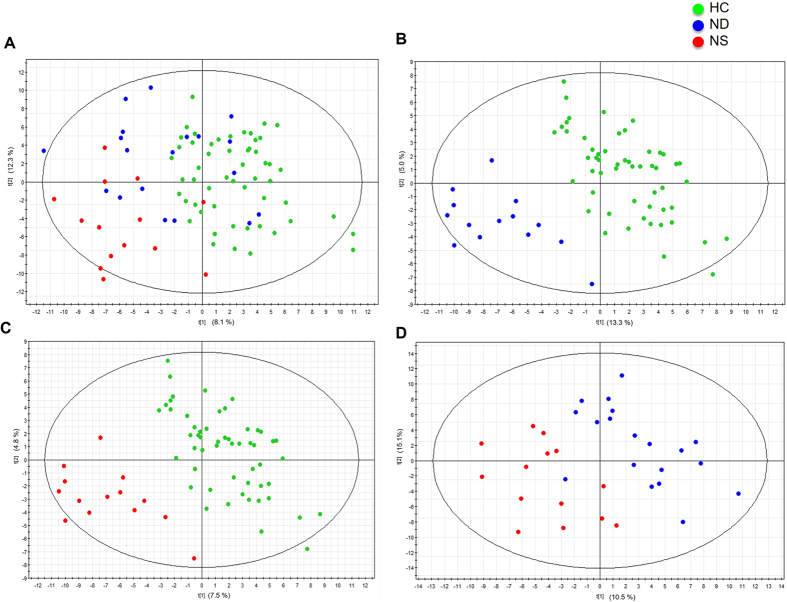
PLS-DA score plots based on DGGE profiles distinguishing between the fecal microbial communities in nonobese individuals with and without NAFLD. (**A**) NAFLD patients vs. HCs, (**B**) ND vs. HCs, (**C**) NS vs. HCs, and (**D**) ND vs. NS. HCs (n = 55), healthy controls; ND (n = 20), individuals with NAFLD; NS (n = 14), NAFLD patients with elevated serum liver enzymes.

Compositional differences in the microbiota of this nonobese cohort were first assessed by DGGE patterns (Supplementary Figure S1A) combined with several clustering methods. As shown in Supplementary Figure S1B, according to multidimensional scaling analysis, an unconstrained clustering method, microbiota composition differed significantly between the two groups. Moreover, PLS-DA (Fig. 1A–C and supplementary Table S1), an supervised analysis method, also allowed for the detection of differences between individuals with and without NAFLD independent of their ALT level, indicating distinctive fecal microbial communities between HC and NAFLD groups. Furthermore, PLS-DA showed that NAFLD individuals with or without normal liver enzyme levels were also associated with clearly different microbiota (Fig. 1D). The validation plot (Supplementary Figure S2) demonstrated that the PLS-DA models were valid: the Q^2^ regression line had a negative intercept, and all of the permuted R^2^ values to the left of the intercept were lower than the original point to the right. These findings suggested the role of gut microbiota in NAFLD and the potential of using the gut microbiota as a marker to differentiate among individuals with or without NAFLD.

### Quantification of fecal microbial changes associated with the presence of NAFLD by 454 pyrosequencing

**Figure 2:**
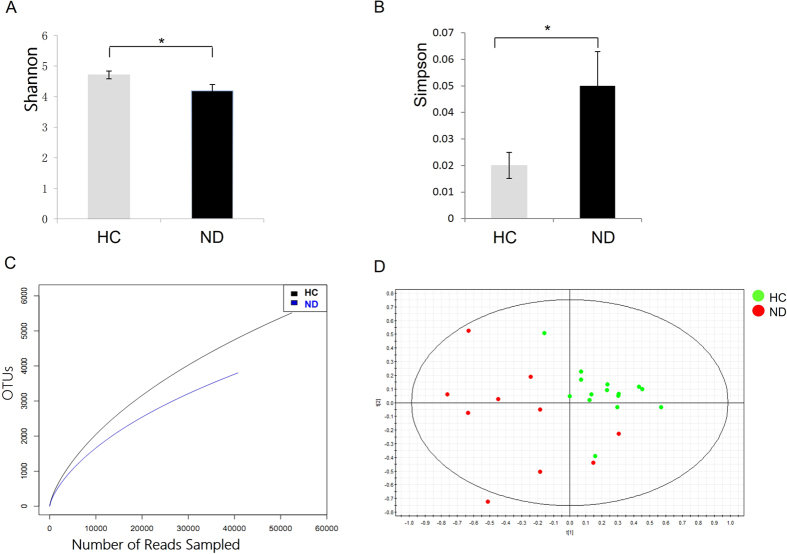
The 454 pyrosequencing analysis revealing decreased ecological diversity of the fecal microbiome in nonobese individuals with NAFLD patients (n = 10) compared with individuals without NAFLD (n = 15). (**A**) The Shannon diversity index, (**B**) the Simpson diversity index, and (**C**) the rarefaction curve trend between patients with NAFLDs and HCs; (**D**) PLS-DA score plots based on the relative abundances of microbial genera of the first two components (t [1] = 20.9%, t [2] = 16.1%) showed the trend that the two groups were well separated.

**Figure 3:**
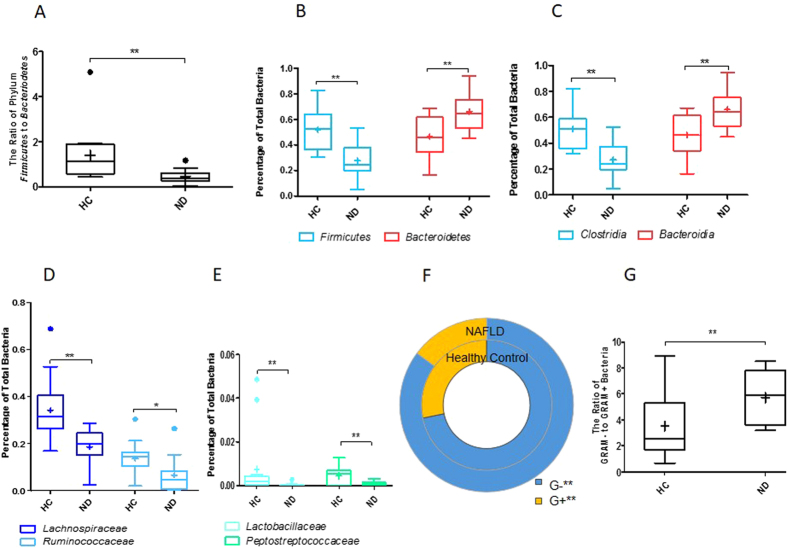
The relative abundances of fecal microbiota significantly differed in individuals with and without NAFLD in the nonobese cohort. (**A**) Ratios of the phyla *Firmicutes* and *Bacteroidetes*, (**B**) phyla *Firmicutes* and *Bacteroidetes*, (**C**) classes clostridia and *Bacteroidia*, (**D**) families *Lachnospiraceae* and *Ruminococcaceae*; (**E**) *Lactobacillaceae* and *Peptostreptococcaceae*; (**F**) G+ and G− bacteria; (**G**) The ratios of G− to G+ bacteria.

To assess quantitative changes in the fecal microbiota associated with the presence of NAFLD, parallel pyrosequencing was performed in a selected subgroup of the nonobese cohort. The demographic and clinical information of the selected subjects and the characteristics of pyrosequencing data were listed in Supplementary Tables S2 and S3, respectively. The distribution of BMI, waist circumference, gender, smoking, alcohol consumption, and dietary habits were not statistically different between individuals with and without NAFLD in the subgroup. In agreement with the findings of DGGE profiling, decreased ecological diversities and altered fecal microbiome compositions of NAFLD patients (ND, n = 8; NS, n = 2) were exhibited compared with those of HCs (n = 15) (Figs 2 and 3 and Supplementary Table S3). As shown in Fig. 2A, the Shannon diversity index (H) was significantly reduced in the NAFLD group (4.19 vs. 4.71, *p* = 0.03), whereas the Simpson’s diversity index (0.05 vs. 0.02, *p* = 0.02) was increased (Fig. 2B), suggesting decreased diversity of the IM in patients with NAFLD. Reduced richness in bacterial diversity was also observed in individuals with NAFLD by the rarefaction curve trend (Fig. 2C). Additionally, the Good’s coverage of each sample for genera was greater than 80%, demonstrating that these sequences may represent major constituents in the bacterial community. Based on the pyrosequencing data, the fecal microbial communities of individuals with or without NAFLD showed clear separation in the PLS-DA score plot (R^2^X = 0.466, R^2^Y = 0.76, Q^2^(cum) = 0.239) (Fig. 2D), indicating that NAFLD was associated with the significant changes in diversity and composition of the fecal microbial community compared with those in HCs.

Further analysis of microbiome data showed that NAFLD was associated with changes in the fecal microbiota at the phylum, class, family and genus levels (Fig. 3, Table 2 and Supplementary Figure S3). As shown in Fig. 3A, a significant decrease in the ratio of *Firmicutes* to *Bacteroidetes* (F/B) (0.47 vs. 1.4, *p* = 0.003) was found between the groups. In addition, individuals with NAFLD had higher fecal levels of the phylum *Bacteroidetes* (66.0% vs. 46.4%, *p* = 0.005) and lower levels of *Firmicutes* (27.5% vs. 51.9%, *p* = 0.002) compared with HCs (Fig. 3B). Furthermore, the predominant class *Bacteroidia* in the phylum *Bacteroidetes* was enriched in patients with NAFLD (65.93% vs. 46.18%, *p* = 0.004), whereas the class *Clostridia* within the phylum *Firmicutes* was depleted in individuals with NAFLD (27.02% vs. 50.91%, *p* = 0.001) (Fig. 3C). Furthermore, the marked depletion of *Firmicutes* in NAFLD group was mostly explained by decreases in four families: *Lachnospiraceae* (18.67% vs. 34.26%, *p* = 0.002), *Ruminococcaceae* (6.69% vs. 13.67%, *p* = 0.018), *Lactobacillaceae* (0.04% vs. 0.73%, *p* = 0.0008), and *Peptostreptococcaceae* (0.09% vs. 0.44%, *p* = 0.002) (Fig. 3D,E).

Moreover, at the genus level, 14 key microbial genera were identified as key microbes with PLS-DA. These genera were responsible for the separation between non-obese individuals with and without NAFLD (Table 2), and well-known gut bacteria such as *Bacteroides*, *Pseudobutyrivibrio* and *Lactobacillus*. Interestingly, differences in the fecal microbiota of individuals with NAFLD were observed only in genera belonging to *Firmicutes* (Table 2 and Supplementary Figure S3). For example, within the *Lachnospiraceae* family, *Coprococcus* (mean 0.34% vs. 2.4%, *p* = 0.009), *Pseudobutyrivibrio* (0.45% vs. 2.16%, *p* = 0.02), *Moryella* (0.05% vs. 0.35%, *p* = 0.02), *Roseburia* (1.48% vs. 2.59%, *p* = 0.01) and *Anaerosporobacter* (1.08% vs. 2.02%, *p* = 0.02) were decreased in individuals with NAFLD. Within the family *Ruminococcaceae*, a similar decrease was observed for *Anaerotruncus* (0.19% vs. 0.96%, *p* = 0.004) and *Ruminococcus* (1.03% vs. 1.59%, *p* = 0.02). Notably, *Lactobacillus*, the main genus of the *Lactobacillaceae* family, was markedly reduced in both abundance (0.04% vs. 0.74%, *p* = 0.003) and prevalence (30% vs. 82%, *p* = 0.008) in NAFLD patients compared with HCs (Supplementary Figure S4). Furthermore, to explore the potential role of the IM in the pathogenesis of NAFLD, the relative abundances of G- bacteria and Gram-positive (G+) bacteria based on the pyrosequencing data were assessed. Importantly, in individuals with NAFLD compared with HCs, G- bacteria were markedly enriched (85.21% vs. 71.8%, *p* = 0.009), and G+ bacteria were decreased (14.79% vs. 28.2%, *p* = 0.01) (Fig. 3F). Additionally, the ratios of G−/G+ bacteria in individuals with NAFLD were enriched compared with those in HCs (5.72 vs. 3.52, *p* = 0.009) (Fig. 3G).

### Potential correlation between intestinal dysbiosis and liver biochemical markers

**Figure 4:**
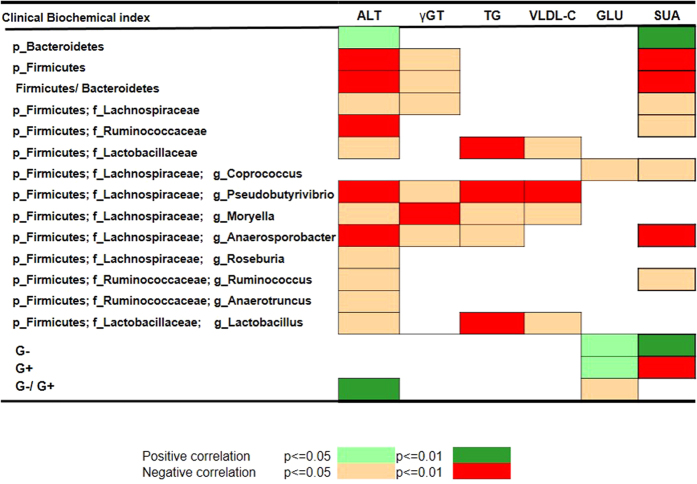


We also attempted to assess the correlation between differences in microbial composition and clinical liver biochemical markers of NAFLD in the non-obese cohort. Interestingly, a clear significant correlation with the set of liver biochemistry indices in plasma was found for NAFLD-induced disturbed microbiota at the phylum and family and genus level by Spearmen’s rank correlation analysis (Fig. 4 and Supplementary Figures S5 and S6). These liver biochemistry markers are well-known clinical biochemical markers for NAFLD diagnosis^1^,^2^,^22^. At the global level, significant correlations with markers of liver status, such as ALT and uric acid, were found for the two dominant phyla *Firmicutes, and Bacteroidetes.* In addition, a significant correlation with γ-GT, markers of liver injury, was also found for *Firmicutes*. Additionally, at the family level, further significant correlations were found for plasma ALT with 3 families (*Lachnospiraceae*, *Ruminococcaceae*, *Lactobacillaceae*), and for plasma γ-GT with *Lachnospiraceae*. Moreover, both plasma total triglycerides and very low-density lipoprotein cholesterol were significantly correlated with *Lactobacillaceae* (*p* < 0.0001).

Furthermore, the correlations between the set of liver biochemistry markers and disturbed microbial genera were also revealed (Fig. 4 and Supplementary Figure S7). For instance, one metabolic marker, fasting glucose, revealed only a single microbial association with *Coprococcus*. Other markers had multiple correlations. For example, the increase of plasma ALT was statistically linked with seven depleted genera (*Pseudobutyrivibrio*, *Moryella*, *Anaerosporobacter*, *Roseburia*, *Ruminococcus*, *Anaerotruncus*, and *Lactobacillus*). These findings indicated a close interrelationship between the intestinal microbial composition and liver biochemical state, highlighting that alterations of the IM are implicated in the presence and development of NAFLD.

## Discussion

To our knowledge, this is the first study assessing the gut microbiota in nonobese individuals with NAFLD compared with those without NAFLD and specifically correlating the microbial composition of individuals with clinical indices. Here, we identified the significant distinguishing fecal microbiota at all taxonomic levels in non-obese individuals with NAFLD compared with those without NAFLD; the former were characterized by a more pronounced *Firmicutes-*poor microbiota and marked lower overall microbial richness. Further analyses revealed that the altered microbiota associated with the presence of NAFLD significantly correlated with liver biochemical markers in the nonobese cohort.

Classifying NAFLD individuals based on BMI is crucial for deciphering the potential role of gut microbiota in NAFLD, because BMI represents a major determinant of compositional changes in microbial communities^25^. Moreover, non-obese patients with NAFLD also represent a considerable portion of NAFLD patients. Notably, in spite of the lower BMI and lower prevalence of obesity in the Asia-Pacific region, people in this region still tend to develop NAFLD. Because both NAFLD and obesity are believed to be influenced by the gut microbiota, comparisons between non-obese individuals with and without NAFLD allowed us to further address the effects of the IM on the liver function.

In human IM, *Firmicutes* and *Bacteroidetes* are the dominant bacteria, accounting for approximately 99% of the whole microbiota^6^,^8^. In our cohort, a significant lower abundance of *Firmicutes* and a higher abundance of *Bacteroidetes* were exhibited in individuals with NAFLD compared with those without NAFLD. This finding was partly in agreement with the findings of Zhu *et al*. and Schwiertz A *et al*., who have demonstrated lower *Firmicutes* and higher *Bacteroidetes* abundances in obese patients compared with lean healthy controls^18^,^26^. But Zhu *et al*. did not find the similar difference between obese and NASH microbiome^18^. The novelty of our study is that we provide the first report of a direct association between lower *Firmicutes* and NAFLD in non-obese individuals. Interestingly, our findings were significantly different with those of a recent report of Mouzaki *et al*., who have shown lower *Bacteroidetes* levels in NASH patients with higher BMI than in patients with simple steatosis and controls with lower BMI^16^. These results indicated that obese NASH and nonobese NAFLD have different fecal microbiome. And the discrepancy between the findings might partly reflect the imbalance of uncontrolled effects of factors between the groups, such BMI and different detection techniques. The decreased proportion of *Bacteroidetes* has also been demonstrated in patients with high BMI in previously published literature in the field of obesity^8^,^9^. Thus, the high BMI of subjects may have affected the results of the study by Zhu *et al*., although the linear regression, adjusting for BMI, was performed to theoretically limit the potential confounding effect of BMI. In addition, the different detection techniques also affected these results between the two study cohorts, which was evidenced by previous studies by our group^11^ and Larsen *et al*.^27^. In our study cohort, all recruited individuals were non-obese adults, which could avoid the confounding factor of BMI. It should be noted that in our non-obese cohort, our observations suggested that a slight increase in BMI, even within the normal range, in people with BMI <25 kg/m^2^ was significantly associated with an increased risk of NAFLD. However, it is unlikely that there are significant BMI-dependent variations in the IM composition within the nonobese spectrum^16^,^18^. And in our quantification study of fecal microbial change, we only selected the nonobese individuals with the median value of BMI to ensure the similar distribution of BMI and waist circumference between two groups. In addition, unbiased metagenomics approaches were applied in this study to characterize the whole IM, including some unknown but potentially functional bacteria^28^.

Bacterial phylotypes decrease in nonobese patients with NAFLD was mostly associated with *Firmicutes*. The families *Lachnospiraceae* and *Ruminococcaceae* are two predominant members of *Firmicutes*. These bacteria are short-chain fatty acids (SCFAs)-producing bacteria that are well known for their health-promoting functions, including the production of nutrients for the host and an energy supply for the colonic epithelium, the modulation of colonic pH^29^, and the maintenance of host immune homeostasis^30^. A decrease in SCFAs might result in deteriorated intestinal integrity and increased intestinal permeability, thus making decreased SCFAs a very important pathogenic factor in NAFLD^31^. In addition, these bacteria are also capable of performing 7-α dehydroxylation and deconjugation in bile acid metabolism, which have been demonstrated to play a critical role in the pathogenesis of chronic liver diseases such as inflammation, cirrhosis and hepatocellular carcinoma^12^,^32^. Indeed, in Jone’s metagenomics study, the function bile salt hydrolases had been identified in all major bacterial phyla and archaeal species in the gut^33^, supporting that IM might modulate the bile acid metabolism in NAFLD patients. In addition, it has been evidenced that IM could modulate the fecal bile acids profiles in patients with cirrhosis^32^ and even promote hepatocellular carcinoma development through modulating the enterohepatic circulation of BAs^12^. Furthermore, we showed that fecal microbial ecological diversity was significantly reduced in patients with NAFLD. Thus far, the exact reason for decreased bacterial diversity in NAFLD remains unclear. It has been demonstrated during antibiotic treatment^34^ and in diseases such as liver cirrhosis^19^ and obesity^35^. Such alterations in the IM have recently been reported for a variety of liver diseases^19^,^32^,^36^. Thus, our finding of general dysbiosis in NAFLD raises the possibility that the compositional change could result in imbalanced microbial ecology, which might itself play a role in increasing the susceptibility to liver diseases.

In this study, we also found a higher abundance of lipopolysaccharide-producing G- bacteria in non-obese individuals with NAFLD. In agreement with our study, through pyrosequencing analysis, De Minicis *et al*. have also revealed an increase in G- bacteria in mice with bile duct ligation fed a high-fat diet^37^. And a significant difference in *Escherichia* was exhibited between obese children with and without NASH in the study by Zhu *et al*.^18^. Additionally, evidence from the literature supports a causal role for endotoxin produced by G- bacteria in NAFLD/NASH^14^,^38^,^39^. Here, a significant positive correlation between G bacteria and fasting glucose levels was also found, highly suggesting the potential contribution of predictive markers for assessing the presence and development of NAFLD in non-obese individuals. It would be interesting to assess differences in the circulating levels of endotoxin between individuals with and without NAFLD in a nonobese cohort.

Notably, we first demonstrated that the health-promoting *Lactobacillaceae* family and its most abundant genus *Lactobacillus* showed marked reductions in both abundance and prevalence in patients with NAFLD compared with HCs. Probiotic bacteria such as *Lactobacillus* and *Bifidobacterium* promote beneficial effects, likely through anti-inflammatory actions and stabilization of the intestinal barrier, thereby attenuating liver pathologies^40^. As concluded by many clinical studies, these probiotic bacteria reduce features of NAFLD in humans^41^,^42^,^43^ and liver injury in mouse models^44^. For example, some strains of *Lactobacillus* lead to substantial reductions in the levels of ALT in 10 patients with NASH^43^. Consistently with the above findings, a negative correlation between *Lactobacillus* and a wide range of biochemical indices of NAFLD progression, including ALT, and TG, was also found, highly facilitating future efforts to better manipulate the gut microbiota for the treatment of NAFLD.

It is widely accepted that the gut microbiota is crucial for NAFLD^31^, which is considered as the hepatic manifestation of metabolic syndrome. However, to date, little is understood about the molecular host-microbiome interactions that influence host metabolic pathways. Recent studies both in adult patients with NAFLD^45^ and mice^13^ supported that gut dysbiosis exerts a critical influence in the progression of NAFLD. In this study a notable finding was the clear correlation between the microbiome composition and well-known clinical indices of NAFLD progression, such as higher blood ALT, γ-GT, TG, VLDL-C, GLU and SUA, suggesting that the microbiota might be closely involved in the pathogenesis of the progression of NAFLD. For example, the blood level of ALT and γ-GT, the inflammatory indices, were significantly correlated with the distinguishing bacteria between individuals with and without NAFLD. In consistent with our findings, Boursier *et al*. also demonstrated that gut dysbiosis associated with NAFLD severity is accompanied by a shift in the metabolic functions of gut microbiota^45^. In addition, the correlation between IM and SUA was also firstly revealed in this study. The level of SUA is elevated significantly in patients with NAFLD, which is an independent risk factor of NAFLD^46^. Uric acid is the major end product of purine metabolism, and has been proposed as a natural scavenger of peroxynitrite and peroxynitrite-derived radicals^47^,^48^. Despite the fact that it is not clear how IM regulates these opposing roles of uric acid on redox balance, our study added new clues that IM might promote steatosis to steatohepatitis in NAFLD by its metabolic pathways and products.

A considerable number of studies support the very important role of the microbiome in the pathogenesis of metabolic disturbance in NAFLD^18^,^30^,^35^,^49^,^50^. Recently, Mouzaki *et al*. have reported a potential negative association between *Bacteroidetes* and insulin resistance when controlling for BMI^18^. Compared with previous reports, our study presents stronger evidence supporting these findings, because we did assess differences in the gut microbiota between nonobese individuals with and without NAFLD; additionally, our study suggests a potential correlation between alterations in fecal microbiota and metabolic disturbance in NAFLD. Future studies are required to establish the mechanistic relevance of our novel findings to the pathogenesis of NAFLD. For instance, it will be necessary to reveal the presence of gut-derived metabolites (i.e., lipopolysaccharide or SCFAs) in the peripheral or portal blood as a medium for the systemic effects of these compounds in human beings or gut microbiome biomarkers associated with the progression of NAFLD and/or NASH.

There were several limitations in this study. First, NAFLD was diagnosed by ultrasonographic methods, which could not determine the severity of NAFLD^51^. Nevertheless, ultrasonography is reported with reasonable accuracy and widely used for population-based studies^22^,^51^. Here, all included subjects had no history of any disease and were recruited at the time of their annual physical examination, it was impractical and unethical to perform liver biopsy in this study. And we performed all the ultrasonographic diagnosis by a single experienced radiographer and the diagnostic performance of the ultrasonographer was evaluated by one senior ultrasonographer meanwhile. Second, this study design did not allow for the examination of insulin resistance, although limited research has found insulin resistance might be a risk factor of NAFLD among nonobese individuals^23^. Future studies are necessary to investigate the correlation between insulin resistance and IM in nonobese NAFLD. Despite these limitations, our conceptually novel study provided important insights into the IM in nonobese adult patients with NAFLD and may have significant clinical importance for NAFLD manipulation.

In summary, our findings firstly showed that the non-obese patients with NAFLD were characterized by a decrease in gut microbial diversity, the depletion of *Firmicutes* including health-promoting bacteria such as SCFAs-producing *Lachnospiraceae,* 7α-dehydroxylating *Ruminococcaceae* and beneficial *Lactobacillaceae*, and the increase of potentially opportunistic pathogenic lipopolysaccharide-producing bacteria. In addition, we established a link between the altered microbiota and well-known liver biochemical indices of NAFLD progression. This novel study may pave the way for new prevention and therapeutic principles. To establish clinical feasible diagnostic and therapeutic methods, further studies utilizing the integrative approach of metabonomics and metagenomics with computational technology^6^ are required to elucidate the key microbes and its metabolic pathway associated with NAFLD.

## Methods

### Patients and study design

The study protocol was approved by the Ethical Review Board of the first affiliated hospital of the medical school of Zhejiang University and conformed to the ethical guidelines of the 1975 Declaration of Helsinki. Volunteer subjects were randomly recruited during their annual health survey. Exclusion criteria included the following: BMI ≥25 kg/m^2^; excess alcohol consumption (>20 g per day for men and >10 g per day for women); a history of cancer, respiratory problems, renal diseases, or endocrine disorder; a history of viral hepatitis, autoimmune hepatitis, genetic hemochromatosis, primary biliary cirrhosis, primary sclerosing cholangitis, α1-antitrypsin deficiency, Wilson’s disease, drug-induced hepatotoxicity, or other known causes of chronic liver disease; an intake history of any medicine, antibiotics or probiotics within the preceding 3 months; and a history of prescription medications, dietary restrictions or lifestyle modifications through diet and exercise at enrollment. Written informed consent and individual information were obtained from all of the volunteer subjects. Data on cigarette smoking, alcohol consumption, physical activity, dietary habits, and a family history of hypertension, diabetes or NAFLD were obtained through a questionnaire. A positive smoking history was defined as currently smoking or with a history of chronic/regular smoking^52^. The positive physical activity habit was defined as having at least 120 min exercise per week^53^. All subjects were required to avoid strenuous physical exercise and high-energy food with the 24 hours preceding the examination and sampling.

Clinical physical examinations were performed in all enrolled subjects by trained medical staff using a standardized procedure. Briefly, a detailed medical history and health habit inventory were taken by a physician. Then, physical examination, anthropometric measurements, abdominal (including hepatic) ultrasonic examination, and clinical biochemical measurements were performed. The diagnosis of NAFLD was made based on evidence of fatty liver upon ultrasonography and the exclusion of other known etiologies of chronic liver disease in accordance with the established clinical and ultrasound criteria^54^. And the individuals with diagnosis of metabolic diseases such as diabetes, hypertension and dyslipidemia were excluded. Finally, a total of 126 nonobese subjects were recruited. The microbial community was profiled by DGGE in all subjects (43 NAFLD, 83 HCs) and further examined by 454 pyrosequencing in the sub-cohort (10 NAFLD, 15 HCs).

Hepatic ultrasonic examination was conducted using a Sequoia 512 Acuson sonography machine with a 1.5-MHz probe (Siemens Healthcare, Erlangen, German) and performed by two experienced ultrasonographer, who were blinded to the study design and clinical data. The ultrasound criteria for the diagnosis of fatty liver were based on the suggestions by the Chinese Liver Disease Association^55^. Briefly, fatty liver was defined as a diffuse enhancement of the near-field echo in the hepatic region and gradual attenuation of the far-field echo if combined with any of the following: (1)unclear display of intrahepatic lacuna structure; (2)mild to moderate hepatomegaly with a round and blunt border; or (3)color Doppler ultrasonography showing a reduction in the blood flow signal in the liver or a blood flow signal that is difficult to display even when the distribution of blood flow is normal^22^.

Fresh fecal samples were placed in sterile tubes under anaerobic conditions (GENbox anaer, BioMérieux), transferred to the laboratory immediately in an ice box, and stored at −70 °C after preparation within 15 min^6^. Blood samples collected early in the morning on the same day were collected and sent to the diagnostic testing laboratory for detection of the twenty-two clinical biochemical parameters. Laboratory tests included ALT, aspartate aminotransferase, albumin, platelet count, γ-GT, fasting glucose, and the lipid profile (cholesterol and triglycerides). NAFLD patients with elevated serum liver enzymes (ALT and γ-GT) were highly suspected of patients with NASH^56^.

### Fecal microbial DNA Extraction

Fecal genomic DNA was isolated from stool samples by using a QIAamp DNA Stool Mini Kit (Qiagen, Valencia, CA) according to the kit protocol with modifications, as previously described by us^6^. Briefly, the fecal samples were lysed by incubating the samples in lysis buffer, and this was followed by bead beating with zirconium beads (0.1 mm) with a FastPrep instrument (MP Biomedicals, Carlsbad, CA) for 60 seconds at the speed of 4 m/s, purified with spin columns, and eluted with 200 μl of buffer AE. The quality of extracted genomic DNA was checked by using the 0.8% agarose gel, and the DNA concentrations were determined with a Nano-Drop 1000 spectrophotometer (NanoDrop Technologies). The extracted DNA was stored at −20 °C.

### PCR-DGGE profiling

The dominant gut bacterial community structure from the fecal microbiota was illustrated by PCR-DGGE analysis, as previously described by us^6^. Isolated fecal DNA was used as a template for amplification of the 16S rRNA-V3 region using a universal primer and the hot-start touchdown protocol described by Muyzer *et al*.^57^. The reaction mixture contained 1 unit of TaKaRa (Takara, Dalian, China) rTaq polymerase, 2.5 μl of 10 × PCR buffer, 2 μl of dNTP mixture (2.5 mM), 0.3 μl of each primer (20 pmol/μl), and 20 ng of extracted fecal DNA in a total volume of 25 μl. Reactions were run in a Veriti 96-well thermal cycler (Applied Biosystems, Foster City, CA). To reduce PCR bias, two separate PCR reactions of each sample were pooled for DGGE profiling. The quality of PCR product was checked by a 1% agarose gel and a NanoDrop 1000 spectrophotometer (NanoDrop Technologies). PCR-DGGE fingerprinting was performed using a D-code system (Bio-Rad, USA) with a house-made pipeline, as previously reported^24^. The DGGE images and their profiles were analyzed by using Bionumerics software (Applied Maths, Belgium), including multidimensional scaling, which is a grouping technique that allows for a convenient visual interpretation. The intensity and position of bands in each lane in the DGGE image were digitalized into a spectrum of 832 variables. In addition, the acquired spectra were linearly aligned, in which the bands in the marker lane were selected as the standard to rectify mobile shifts of the bands in different DGGE profiles. Then, multivariate data analysis, including principal component analysis and partial least-squares-latent structure discriminate analysis (PLS-DA), was performed by using Simca-*P* 12.0 (Umetrics AB, Sweden) on the data set to observe the structure of the fecal DGGE profiles, such as clustering and outliers^6^,^58^.

### Pyrosequencing and data analyses

Pyrosequencing technology is a powerful tool for the comprehensive analysis of IM^28^ in patients with liver diseases such as liver cirrhosis^11^ and NASH^18^. As previously described, after PCR products amplified and quantified, equimolar concentrations were pooled and sequenced on a 454 Life Sciences Genome Sequencer FLX system (Roche) according to the manufacturer’s recommendations^11^. All reads were deposited in GeneBank (SRA056280), quality trimmed, analyzed and grouped into operational taxonomic units at a sequence similarity level of 97%. These operational taxonomic units were used for diversity (Shannon, Simpson), richness (Chao 1), and rarefaction curve analysis. And bacterial coverage for each library was calculated by Good’s coverage^11^.

The table of normalized genera counts was fed into Simca-*P* 12.0 for principal component analysis and PLS-DA^11^,^59^. Prior to multivariate data statistics analysis, the normalized data were pareto-scaled. And the supervised models were validated with a permutation test that was repeated 200 times to prevent model overfitting^58^. In addition, the variable importance in projection (VIP) index was used to select the discriminating bacteria and reflected the influence of each microbial genus in the different group. Variables with VIP > 1 are important contributors to generation of the model. The Mann-Whitney U test was used to evaluate group differences, and Spearman’s correlation coefficients were used to assess bivariate relationships between variables by using SPSS Version 16.0 (SPSS Inc., Chicago, IL) and GraphPad Prism 5 (GraphPad Software, Inc.). And *p* values had been corrected for multiple testing by using Bonferroni correction.

## Additional Information

**How to cite this article**: Wang, B. *et al*. Altered Fecal Microbiota Correlates with Liver Biochemistry in Nonobese Patients with Non-alcoholic Fatty Liver Disease. *Sci. Rep.* **6**, 32002; doi: 10.1038/srep32002 (2016).

## Supplementary Material

Supplementary Information

## Figures and Tables

**Table 1:** Demographic, anthropometrics and laboratory data of the study cohorts.

Variables	With NAFLD (n = 43)	Without NAFLD (n = 83)	*p*-value
Sex (male/female)	43 (36/7)	83 (70/13)	—
Age (years)	47.0 (34.5–61.0)	40.5 (33.0–52.0)	0.31
Body mass index (kg/m^2^)	23.19 (22.19–24.22)	21.77 (20.7–23.38)	0.004
Waist circumference (cm)	89 (84–91.75)	79.5 (72.5–85)	<0.0001
Hip circumference (cm)	98 (94.75–100)	95 (91–97)	0.0058
Waist-to-hip ratio	0.90 (0.89–0.92)	0.84 (0.80–0.89)	<0.0001
Systolic blood pressure (mm Hg)	127.0 (111.5–136.0)	119 (110–129)	0.19
Diastolic blood pressure (mm Hg)	75 (70.5–82.5)	72 (67–80)	0.36
ALT (U/l)	29 (20.5–39.5)	14.5 (12–20.75)	0.0001
AST (U/l)	22 (18–25.5)	20 (18.0–21.75)	0.06
Alkaline phosphatase (g/l)	78 (63–91)	65 (54–78.75)	0.002
Total bilirubin (μmol/l)	13 (11–18)	12 (10–17)	0.182
Direct bilirubin (μmol/l)	4 (3–5)	4 (3–5)	0.95
Indirect bilirubin (μmol/l)	10 (8.5–13)	9 (6.25–12)	0.12
Total triglycerides (mmol/l)	1.83 (1.43–2.6)	1.04 (0.77–1.45)	0.0001
Total cholesterol (mmol/l)	4.9 (4.25–5.36)	4.68 (4.1–5.11)	0.12
High-density lipoprotein cholesterol (mmol/l)	1.12 (0.89–1.30)	1.4 (1.19–1.59)	0.0003
Low-density lipoprotein cholesterol (mmol/l)	2.54 (2.14–2.95)	2.50 (2.04–2.79)	0.40
Very low-density lipoprotein cholesterol (mmol/l)	1.01 (0.84–1.42)	0.7 (0.47–0.94)	<0.0001
Fasting glucose (mmol/l)	4.96 (4.74–5.47)	4.87 (4.40–5.20)	0.132
γ-Glutamyltransferase (U/l)	37 (23.5–62.5)	20 (14–28)	<0.0001
Serum uric acid (μmol/l)	383 (310–423)	340.5 (392.3–387.3)	0.016
Hemogobin (g/l)	158 (153–165)	154 (144–164)	0.07
Platelet count (×10^9^/l)	214 (183–250)	199 (168.5–234)	0.08

Note: Data are expressed as the mean (SEM.) or median (IQR, interquartile range).

**Table 2:** The average abundance and the variable importance in projection index of the key differentiating genera between non-obese individuals with and without NAFLD identified by the PLS-DA model based on the 454 pyrosequencing data.

NO.	Genus	Family	Phylum	VIP	With ND	Without ND	*p*value
1	*Bacteroides*	*Bacteroidaceae*	*Bacteroidetes*	5.9	29% ± 4%	20% ± 1.8%	0.103
2	*Prevotella*	*Prevotellaceae*	*Bacteroidetes*	3.8	8% ± 4%	3% ± 1%	0.789
3	*Pseudobutyrivibrio*	*Lachnospiraceae*	*Firmicutes*	1.9	0.45% ± 0.2%	2.16% ± 0.6%	0.02
4	*Anaerotruncs*	*Ruminococcaceae*	*Firmicutes*	1.9	0.19% ± 0.2%	0.96% ± 0.4%	0.004
5	*Lactobacillus*	*Lactobacillaceae*	*Firmicutes*	1.7	0.04% ± 0.03%	0.74% ± 0.4%	0.003
6	*Roseburia*	*Lachnospiraceae*	*Firmicutes*	1.6	1.48% ± 0.6%	2.59% ± 0.4%	0.01
7	*Coprococcus*	*Lachnospiraceae*	*Firmicutes*	1.6	0.34% ± 0.1%	2.4% ± 0.6%	0.009
8	*Streptococcus*	*Streptococcaceae*	*Firmicutes*	1.5	0.28% ± 0.01%	0.2% ± 0.1%	0.24
9	*Ruminococcus*	*Ruminococcaceae*	*Firmicutes*	1.4	1.03% ± 0.7%	1.59% ± 0.3%	0.02
10	*Moryella*	*Lachnospiraceae*	*Firmicutes*	1.4	0.05% ± 0.02%	0.35% ± 0.1%	0.02
11	*Anaerosporabacter*	*Lachnospiraceae*	*Firmicutes*	1.4	1.08% ± 0.1%	2.02% ± 0.1%	0.02
12	*Oscillibacter*	*Ruminococcaceae*	*Firmicutes*	1.2	0.8% ± 0.4%	1.4% ± 0.3%	0.05
13	*Bifidobacterium*	*Bifidobacteriaceae*	*Actinobacteria*	1.2	0.36% ± 0.2%	0.22% ± 0.1%	0.82
14	*Escherichia*	*Enterobacteriaceae*	*Proteobacteria*	1	0.32% ± 0.3%	0.1% ± 0.05%	0.78

Note: Data are expressed as the mean (SEM.); VIP, the variable importance in projection.
