# Supplementary material for: Altered Fecal Microbiota Correlates with Liver Biochemistry in Nonobese Patients with Non-alcoholic Fatty Liver Disease
Source: Sci Rep. 2016 Aug 23;6:32002. doi: 10.1038/srep32002 (PMC4994089; doi:10.1038/srep32002)
Supplement: Supplementary Information [file srep32002-s1.doc]

**Title: Altered Fecal Microbiota Correlates with Liver Biochemistry in Nonobese Patients with Non-alcoholic Fatty Liver Disease**

**Authors**: Baohong Wang, Xiangyang Jiang, Min Cao, Jianping Ge, Qiongling Bao, Lingling Tang, Yu Chen, and Lanjuan Li*.

**Supplementary Information**

**Supplementary Tables**

**Supplementary Table S1**. Summary of parameters of model efficiency for assessment of the quality of PLS-DA models based on fecal DGGE profiles of nonobese individuals with and without NAFLD in this study.

**Supplementary Table S2**. Clinical characteristics of the subjects in the pyrosequencing study.

**Supplementary Table S3**. Characteristics of pyrosequencing data.

**Supplementary Figures:**

**Supplementary Figure S1.** (A) The representative DGGE pattern for the predominant fecal bacteria in nonobese individuals with and without NAFLD. Mr, marker lane. (B) Multidimensional scaling (MDS) plot indicating the clear differences in fecal microbiota composition in individuals with and without NAFLD in the nonobese cohorts. MDS was based on the DGGE profiles.

**Supplementary Figure S2.** Validation plot of the PLS-DA models obtained from 200 permutation tests based on fecal DGGE profiles in nonobese individuals with and without NAFLD.

**Supplementary Figure S3**. The reduced average abundance of bacterial genera in nonobese individuals with NAFLD in comparison with individuals without NAFLD.

**Supplementary Figure S4**. The difference in the prevalence of bacterial genus in nonobese individuals with and without NAFLD.

**Supplementary Figure S5**. Significant correlation between the changes in fecal bacterial phylum and clinical biochemistry indices.

**Supplementary Figure S6**. Significant correlation between the changes in fecal bacterial family and clinical biochemistry indices.

**Supplementary Figure S7**. Significant correlation between the changes in fecal genus and clinical biochemistry indices, including (A) ALT; (B) ᵞ-GT; (C) TG; (D) VLDL-C; (E) GLU; and (F) SUA.

**Supplementary Table S1**. Summary of parameters of model efficiency for assessment of the quality of PLS-DA models based on fecal DGGE profiles of nonobese individuals with and without NAFLD in this study.

| Model no. | groups | R^2^X  (cum) | R^2^Y  (cum) | Q^2^  (cum) | component |
| --- | --- | --- | --- | --- | --- |
| 1 | All patients (ND and NS) vs controls | 0.204 | 0.365 | 0.10 | 2 |
| 2 | ND vs controls | 0.322 | 0.823 | 0.126 | 4 |
| 3 | NS vs controls | 0.243 | 0.854 | 0.190 | 3 |
| 4 | ND vs NS | 0.34 | 0.89 | 0.376 | 3 |

**Supplementary Table S2**. Clinical characteristics of the subjects in the pyrosequencing study.

| Variables | Healthy controls (n = 15) | NAFLD patients (n = 10) | *p*_value |
| --- | --- | --- | --- |
| Sex (male /female) | (10/5) | (7/3) | - |
| Smoking (yes /no) | (0/15) | (0/10) | - |
| Dietary habits  (omnivorous /vegetarian) | (15/0) | (15/0) | - |
| Alcohol consumption (yes /no) | (0/15) | (0/10) | - |
| Physical activity (yes /no) | (0/15) | (0/10) | - |
| Age (years) | 37.9 ± 2.23 | 42.5 ± 2.63 | 0.31 |
| Body mass index (kg /m^2^) | 20.8 ± 0.70 | 22.3 ± 1.10 | 0.06 |
| ALT (U/l) | 12.0 ± 1.45 | 28.0 ± 3.79 | 0.001 |
| AST (U/l) | 17.0 ± 1.21 | 21.0 ± 1.90 | 0.14 |
| Total bilirubin (µmol/l) | 14.2 ± 1.19 | 12.0 ± 1.26 | 0.25 |
| Total triglycerides (mmol/l) | 1.18 ± 0.22 | 1.95 ± 0.31 | 0.05 |
| Total cholesterol (mmol/l) | 4.46 ± 0.16 | 5.19 ± 0.16 | 0.17 |
| High-density lipoprotein cholesterol (mmol/l) | 1.38 ± 0.08 | 1.18 ± 0.01 | 0.09 |
| Low-density lipoprotein cholesterol (mmol/l) | 2.24 ± 0.12 | 2.68 ± 0.36 | 0.29 |
| Very low-density lipoprotein cholesterol (mmol/l) | 0.83 ± 0.11 | 1.33 ± 0.15 | 0.03 |
| Fasting glucose (mmol/l) | 4.68 ± 0.07 | 5.20 ± 0.24 | 0.05 |
| γ-Glutamyltransferase (U/l) | 20.3 ± 5.5 | 28.7 ± 6.0 | 0.02 |
| Serum uric acid (µmol/l) | 271.3 ± 20.3 | 349.8 ± 33.6 | 0.02 |

Note: Data are expressed as the mean (SEM.).

**Supplementary Table S3**. Characteristics of pyrosequencing data.

| Diversity index | Healthy controls (n = 15) | NAFLD patients (n = 10) | *p*_ value |
| --- | --- | --- | --- |
| OTUs^1^ | 567 ± 70 | 534 ± 69 | 0.89 |
| Good’s (%)^2^ | 80% ± 2.9% | 83% ± 2.1% | 0.11 |
| ACE | 2187 ± 425.2 | 1874 ± 396.2 | 0.63 |
| Chao 1 | 1394 ± 271.64 | 1207 ± 260.9 | 0.65 |
| Shannon index | 4.71 ± 0.52 | 4.19 ± 0.66 | 0.03 |
| Simpson index | 0.025 ± 0.02 | 0.054 ± 0.04 | 0.02 |

Data are expressed as the mean (SEM.).

^1^ The operational taxonomic units (OTUs) were defined at the 97% similarity level.

^2^ The coverage percentage (Good’s), richness estimators (ACE and Chao1) and diversity indices (Shannon and Simpson) were calculated using Good’s method and the MOTHUR program respectively.

**Supplementary Figure S1.** (A) The representative DGGE pattern for the predominant fecal bacteria in nonobese individuals with and without NAFLD. Mr, marker lane. (B) Multidimensional scaling (MDS) plot indicating the clear differences in fecal microbiota composition in individuals with and without NAFLD in the nonobese cohorts. MDS was based on the DGGE profiles.


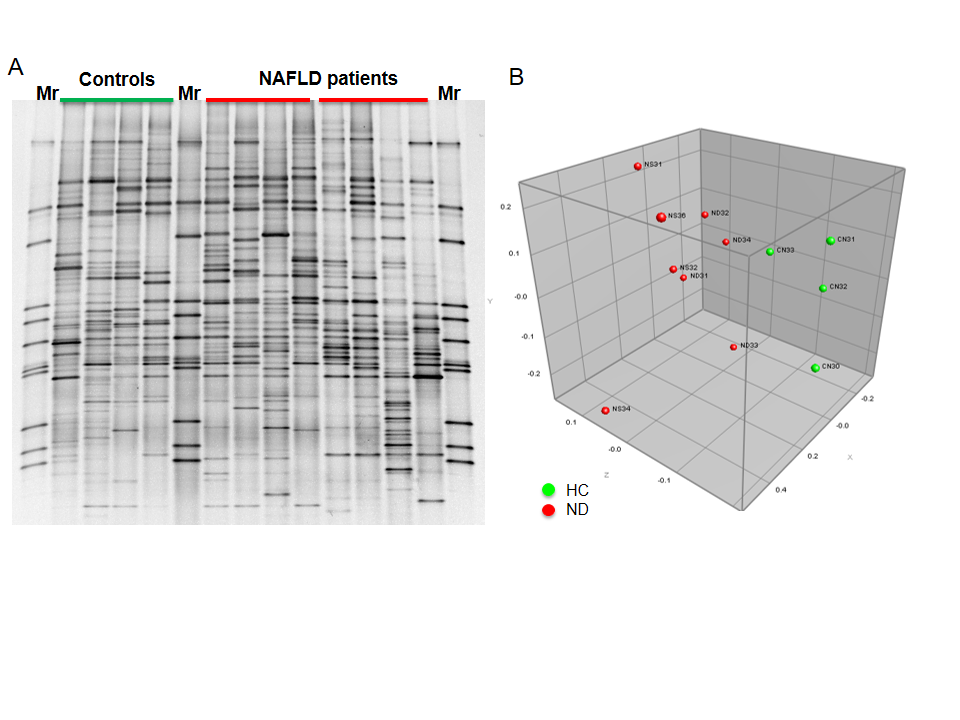


**Supplementary Figure S2.** Validation plot of the PLS-DA models obtained from 200 permutation tests based on fecal DGGE profiles in nonobese individuals with and without NAFLD. (A) Model 1: All patients (ND and NS) vs. HCs; (B) Model 2: ND vs. controls; (C) Model 3: NS vs. controls; (D) Model 4: ND vs. NS.

**
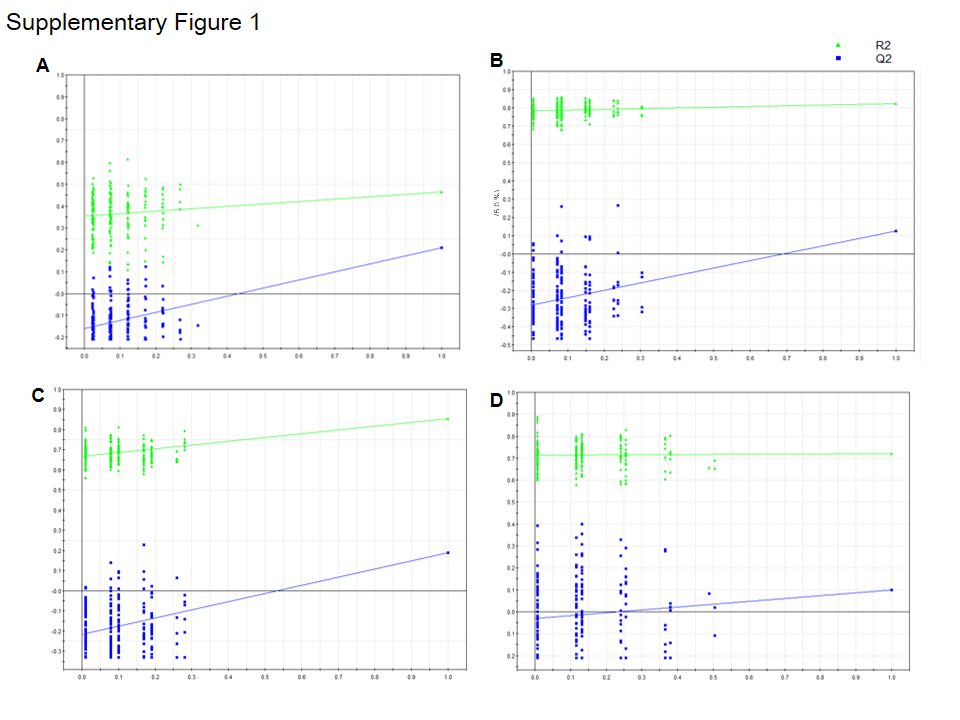
**

**Supplementary Figure S3**. The reduced average abundance of bacterial genera in nonobese individuals with NAFLD in comparison with individuals without NAFLD.

**Supplementary Figure S4**. The difference in the prevalence of bacterial genus in nonobese individuals with and without NAFLD.


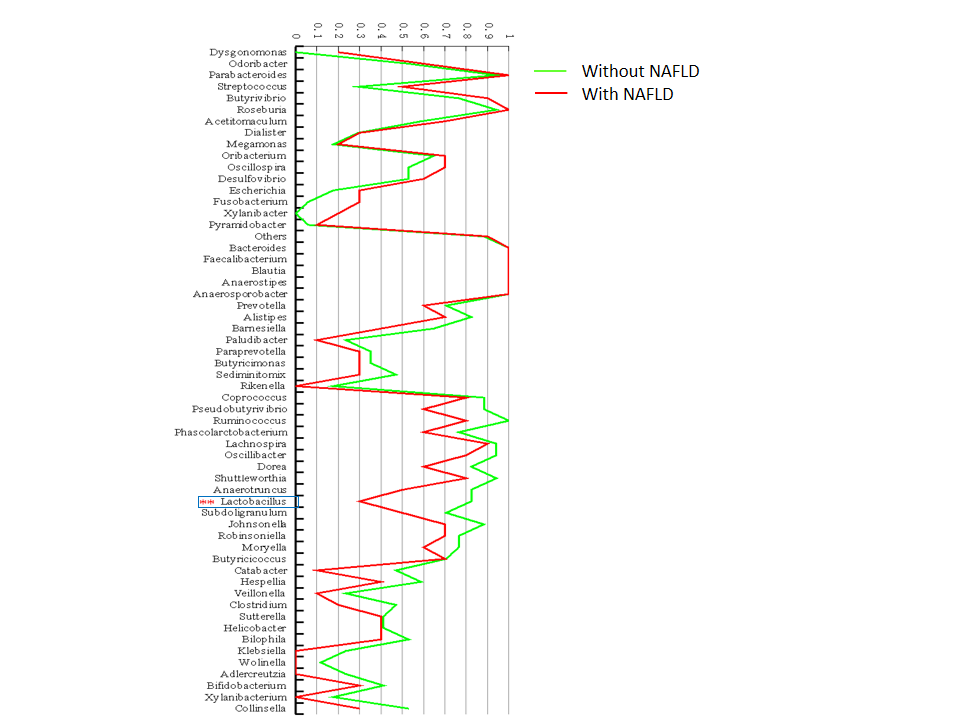


**Supplementary Figure S5**. Significant correlation between the changes in fecal bacterial phylum and clinical biochemistry indices.

**Supplementary Figure S6**. Significant correlation between the changes in fecal bacterial family and clinical biochemistry indices.

**Supplementary Figure S7**. Significant correlation between the changes in fecal genus and clinical biochemistry indices, including (A) ALT; (B) ᵞ-GT; (C) TG; (D) VLDL-C; (E) GLU; and (F) SUA.

(A)

(B)

(C).

(D).

(E).

(F).
